# The Production of Nominal and Verbal Inflection in an Agglutinative Language: Evidence from Hungarian

**DOI:** 10.1371/journal.pone.0119003

**Published:** 2015-03-13

**Authors:** Dezso Nemeth, Karolina Janacsek, Zsolt Turi, Agnes Lukacs, Don Peckham, Szilvia Szanka, Dorottya Gazso, Noemi Lovassy, Michael T. Ullman

**Affiliations:** 1 Institute of Psychology, Eotvos Lorand University, Budapest, Hungary; 2 Department of Psychology, Georgetown University, Washington, DC, United States of America; 3 Department of Clinical Neurophysiology, University of Göttingen, Göttingen, Germany; 4 Department of Cognitive Science, Budapest University of Technology and Economics, Budapest, Hungary; 5 Department of English Language Teacher Education and Applied Linguistics, University of Szeged, Szeged, Hungary; 6 Institute of Psychology, University of Szeged, Szeged, Hungary; 7 Brain and Language Lab, Department of Neuroscience, Georgetown University, Washington, DC, United States of America; University of Barcelona, SPAIN

## Abstract

The contrast between regular and irregular inflectional morphology has been useful in investigating the functional and neural architecture of language. However, most studies have examined the regular/irregular distinction in non-agglutinative Indo-European languages (primarily English) with relatively simple morphology. Additionally, the majority of research has focused on verbal rather than nominal inflectional morphology. The present study attempts to address these gaps by introducing both plural and past tense production tasks in Hungarian, an agglutinative non-Indo-European language with complex morphology. Here we report results on these tasks from healthy Hungarian native-speaking adults, in whom we examine regular and irregular nominal and verbal inflection in a within-subjects design. Regular and irregular nouns and verbs were stem on frequency, word length, and phonological structure, and both accuracy and response times were acquired. The results revealed that the regular/irregular contrast yields similar patterns in Hungarian, for both nominal and verbal inflection, as in previous studies of non-agglutinative Indo-European languages: the production of irregular inflected forms was both less accurate and slower than of regular forms, both for plural and past-tense inflection. The results replicate and extend previous findings to an agglutinative language with complex morphology. Together with previous studies, the evidence suggests that the regular/irregular distinction yields a basic behavioral pattern that holds across language families and linguistic typologies. Finally, the study sets the stage for further research examining the neurocognitive substrates of regular and irregular morphology in an agglutinative non-Indo-European language.

## Introduction

Regular and irregular inflectional morphology has been the focus of extensive research in recent decades. Regular forms are generally defined as those that undergo only fully predictable default transformations, including affixation (e.g., *walk-walked* in English past-tense). Irregulars, in contrast, undergo at least partly idiosyncratic transformations, often including stem changes, whether or not the modified stems are also affixed (e.g., *sing-sang*, *keep-kept*). The regular/irregular distinction has been examined in numerous studies using a variety of different approaches, including with behavioral, developmental, neurological, electrophysiological, and neuroimaging methodologies [1,2,3,4,5,6,7,8,9]. The distinction appears to constitute a useful paradigm for examining the psychological, computational, and neural underpinnings of language, even though the exact mechanisms underlying regular and irregular morphology are still not resolved [1–3,55,58–59]. The use of regular and irregular forms may also facilitate the creation of targeted sensitive language tests that could have potential diagnostic value in various neurological and psychiatric disorders (e.g., Huntington’s, Parkinson’s, and Alzheimer’s diseases, carotid stenosis) [10,11,12].

The vast majority of research on regular and irregular inflection has focused on one language, English. However, English has relatively simple inflectional morphology, and represents only one type of morphological system. Traditionally, languages of the world are classified into four major morphological types—although many languages contain elements from different types, and languages lie along a continuum where these types represent extremes. At one extreme are isolating languages (e.g., Chinese), which are characterized by a very small number of affixes, with grammatical relationships mainly being encoded by relative word position. In languages with fusional morphology, such as Italian, Russian, Arabic, or English, there are more affixes, each of which may represent several functions (e.g., in “he walks”, the –*s* affix represents 3^rd^ person singular present tense). In agglutinative languages such as Turkish, Hungarian, or Finnish, words can be composed of multiple affixes, with each being clearly distinguishable and having a unique grammatical or semantic function. Finally, in polysynthetic languages (e.g., Inuit languages), content and function words are strung together into long chains to form very complex word forms representing entire sentences.

Because of these typological differences, it is possible that findings from English might not generalize to other languages. Thus, an increasing number of studies have examined regular and irregular morphology in other languages with more complex morphology, including Spanish [13,14,15], Italian [16,17], German [1,18], Norwegian [19] and Dutch [20]. Even these languages, however, do not broadly cover the typological space, since they all are primarily fusional. Moreover, phylogenetically they are all Indo-European languages. Although some research has probed regular/irregular distinctions in non-Indo-European languages such as Hebrew [21] or Arabic [22], again typologically they are primarily fusional. Thus, the investigation of regular/irregular morphology should be extended to substantially different typological languages, as well as to different language families, to examine whether findings are truly general.

Extending the investigation to agglutinative languages would be an important step, given their morphological complexity. Indeed, the regular-irregular distinction has begun to be examined in some languages with agglutination, such as Japanese [23,24] or Korean [9]. One problem with many agglutinative languages, however, including these two languages, is that they often do not contain many irregular forms, in particular those with stem changes. This limits the power and scope of investigations of regular/irregular contrasts in these languages. Although some studies have addressed this issue by examining default vs. non-default “regular” forms without stem changes [Fujiwara, 2000 #9886; Fujiwara, 1999 #8237], this approach does not further elucidate the regular/irregular contrast itself.

Nevertheless, irregularity can be quite common in some languages with substantial agglutination, such as Hungarian. Of interest here, although in Hungarian, plurals always contain the –*k* suffix, this affix can be appended either in a completely productive way (e.g., *lány-lányok*, girl—girls) or following unproductive patterns for a closed class of words, often involving modifications to the stem (irregular; e.g., *ló—lovak*, horse—horses). A similar regular/irregular contrast exists for past-tense verbal inflection in Hungarian. Thus Hungarian is well-suited for examining the regular/irregular distinction in an agglutinative language. Moreover, Hungarian is not an Indo-European language. Rather it is in the Uralic language family, which also contains Finnish, Estonian and various languages in Russia.

Several previous empirical studies have examined regular and irregular inflectional morphology in Hungarian. Most have used receptive tasks (e.g., priming), though these have been limited to plural inflection (and have all been published in Hungarian, decreasing their accessibility to a broad readership) [25,26,27]. We are aware of four studies that have examined the regular/irregular contrast with production tasks [10,11,28,29,30]. However, all four tested patient groups (Huntington’s disease, carotid stenosis, Williams syndrome, and Specific Language Impairment), and had relatively small (maximum 30) subject numbers for both patients and controls. Moreover, all four examined only plural production, and regulars and irregulars were not well-matched. Finally, these studies measured only accuracy, not response (reaction) times, precluding the assessment of on-line processing of regular and irregular morphology in Hungarian. Although accuracy measures can be revealing, response time measures can be additionally informative, and might be potentially relevant for inferring psycholinguistic processes during the processing of inflectional morphology [31]. Thus important gaps remain in the investigation of regular and irregular morphology in Hungarian, as well as in agglutinative languages more generally.

Here we present new elicited production tasks probing Hungarian regular and irregular morphology in both plural nominal inflection and past-tense verbal inflection. The inclusion of both types of inflection is important, since most previous work on regular/irregular inflection has focused on verbal morphology. The regular and irregular nouns and verbs were matched on stem frequency, word length and phonological structure. Both tasks were given to 85 healthy young adult subjects in a within-subjects design. Both accuracy and response times (RTs) were measured on all items. The study is thus designed to reveal any reliable patterns in the production of regular and irregular inflectional morphology, across word classes, in a non-Indo-European agglutinative language with complex morphology. The study should reveal whether or not, or to what extent, previous inflectional production results obtained in Indo-European non-agglutinative languages hold more generally across language families (in Uralic as well as Indo-European), structural typologies (agglutinative as well as non-agglutinative), and linguistic categories (nouns as well as verbs).

We hypothesized that regular and irregular forms for Hungarian plurals and past tenses should display a similar pattern to that observed for regulars and irregulars in other languages—despite any typological or other differences between the languages—due to the underlying nature of the regular/irregular distinction (see Discussion): that is, an advantage (in accuracy and/or reaction times) for regulars as compared to irregulars [14–15,31,41].

## Materials and Methods

### Ethics Statement

Ethics approval was obtained by the Psychology Ethics Committee at the Institute of Psychology, University of Szeged. All participants provided signed informed consent agreements, and received no financial compensation for their participation.

### Participants

Eighty-five healthy young adults (35 males and 50 females; 71 right- and 14 left-handed) participated in the experiment as volunteers. The mean age was 21.76 years (SD = 2.43). All of them were university students, with a mean of 15.12 years of formal education (SD = 1.79). All participants were native Hungarian speakers with normal or corrected-to-normal vision. No participant had any known psychiatric, neurological or neurodevelopmental disorder.

### Materials

#### Plural Production Task

In Hungarian, the singular form of nouns is represented by the bare stem. Plural forms consist of the stem appended with the plural suffix –*k*. If the stem ends in a consonant, the plural forms contains an extra vowel (-V*k*), where V represents a vowel that harmonizes with one or more of the stem vowels. Hungarian nouns can be categorized into those that take regular or irregular plurals. When regular nouns combine with suffixes, the stem either does not change, if the final stem phoneme is a consonant or a long vowel (e.g., *lapát*—*lapátok*, shovel—shovels; *cipő—cipők*, shoe—shoes), or, if the stem ends with a low vowel (a or e), it changes according to a productive morpho-phonological rule, that is, stem-final vowel lengthening (e.g., *alma—almák*, apple—apples; *csésze—csészék*, cup—cups). This morpho-phonological rule is fully predictable, and applies even to neologisms. In contrast, Hungarian irregular nouns exhibit idiosyncratic morpho-phonological modifications on the stem depending on stem class (v-inserting stems: e.g., *ló*—*lovak*, horse—horses, epenthetic stems: e.g. *bokor*—*bokrok*, bush—bushes; shortening stems: *madár*—*madarak*, bird—birds). These phonological changes are not predictable on the basis of any phonological or semantic features. Thus, irregular plurals are formed not only by combining stems with a suffix (like regulars), but additionally by undergoing unpredictable phonological changes to the stem. In the plural production task developed here, we include only regular nouns with no stem change, and irregular nouns with any of three types of stem changes; see Table 1. All regular and irregular nouns ended in a consonant. For further details on Hungarian morphology, see [32,33].

**Table 1:** Examples of Hungarian regular and irregular noun types used in this study, with singular and plural forms.

	Stem type	Examples
Regular	Stems ending in a consonant(stem + -V*k*)	*lapát*—*lapátok*
		shovel—shovels
	Epenthetic	*kéreg—kérgek*
		bark—barks
Irregular	Shortening	*madár—madarak*
		bird—birds
	‘v’-inserting	*ló*—*lovak*
		horse—horses

The plural production task consisted of 26 regular and 26 irregular nouns (Table 2). The irregulars included the following stem classes: epenthetic, shortening and ‘v’-inserting stem classes (Table 1). The regulars and irregulars were matched pairwise on stem word length (means 1.96 vs. 1.96 syllables, respectively; *t* (25) < 0.001, *p* > .999) and CV (consonant vowel) structure (see Table 2), as well as natural logarithm-transformed bare stem frequency (2.96 vs. 2.98; *t* (25) = −1.53, *p* = .14, respectively) [34,35]. Inflected form frequencies were not available for all nouns, and hence regulars and irregulars were not matched on this factor. Each noun was pseudo-randomly ordered in the presentation list, with the different types relatively evenly distributed throughout the list.

**Table 2:** Regular and irregular nouns in the plural production task.

Stem form	Plural form	Translation	Stem syllable #	Plural syllable #	Stem CV structure	Plural CV structure	Stem frequency (ln-transformed)	Mean Accuracy	SD Accuracy	Mean RT	SD RT
						**REGULAR**					
*búvár*	*búvárok*	diver	2	3	CVCVC	CVCVCVC	3.23	1.00	0.00	752.88	299.69
*ketrec*	*ketrecek*	cage	2	3	CVCVC	CVCCVCVC	2.86	1.00	0.00	710.30	251.95
*betyár*	*betyárok*	rascal	2	3	CVCVC	CVCVCVC	3.16	1.00	0.00	756.91	227.58
*papucs*	*papucsok*	slippers	2	3	CVCVC	CVCVCVC	2.76	1.00	0.00	720.05	262.74
*mókus*	*mókusok*	squirrel	2	3	CVCVC	CVCVCVC	3.17	1.00	0.00	725.59	229.57
*lazac*	*lazacok*	salmon	2	3	CVCVC	CVCVCVC	2.75	1.00	0.00	643.96	206.30
*köteg*	*kötegek*	bundle	2	3	CVCVC	CVCVCVC	2.76	1.00	0.00	655.04	221.07
*kupac*	*kupacok*	pile	2	3	CVCVC	CVCVCVC	2.77	.99	0.11	689.64	251.18
*eresz*	*ereszek*	eaves	2	3	VCVC	VCVCVC	2.76	1.00	0.00	809.81	344.96
*ideg*	*idegek*	nerve	2	3	VCVC	VCVCVC	2.80	1.00	0.00	799.64	359.15
*ámen*	*ámenek*	amen	2	3	VCVC	VCVCVC	2.74	.93	0.26	767.81	323.11
*hólyag*	*hólyagok*	blister	2	3	CVCVC	CVCVCVC	2.87	1.00	0.00	708.35	289.71
*íjász*	*íjászok*	bowman	2	3	VCVC	VCVCVC	2.77	.99	0.11	739.59	313.20
*fűszer*	*fűszerek*	spice	2	3	CVCVC	CVCVCVC	2.93	.98	0.16	746.65	292.95
*vagon*	*vagonok*	wagon	2	3	CVCVC	CVCVCVC	2.95	1.00	0.00	738.08	256.64
*ecset*	*ecsetek*	brush	2	3	VCVC	VCVCVC	2.98	1.00	0.00	670.65	244.02
*fűrész*	*fűrészek*	saw	2	3	CVCVC	CVCVCVC	2.78	1.00	0.00	759.73	419.26
*cseléd*	*cselédek*	servant	2	3	CVCVC	CVCVCVC	2.98	1.00	0.00	718.87	303.77
*báb*	*bábok*	puppet	1	2	CVC	CVCVC	2.98	.99	0.11	691.77	261.11
*haver*	*haverok*	buddy	2	3	CVCVC	CVCVCVC	3.17	.98	0.15	704.70	282.63
*köpeny*	*köpenyek*	cloak	2	3	CVCVC	CVCVCVC	3.07	.99	0.11	657.77	280.82
*bohóc*	*bohócok*	clown	2	3	CVCVC	CVCVCVC	3.15	1.00	0.00	660.11	214.66
*kazán*	*kazánok*	boiler	2	3	CVCVC	CVCVCVC	3.21	.99	0.11	702.59	253.49
*kavics*	*kavicsok*	pebble	2	3	CVCVC	CVCVCVC	3.25	1.00	0.00	593.92	140.01
*csónak*	*csónakok*	boat	2	3	CVCVC	CVCVCVC	3.35	1.00	0.00	681.52	289.55
*törés*	*törések*	fracture	2	3	CVCVC	CVCVCVC	3.34	1.00	0.00	742.73	282.33
						**IRREGULAR**					
*féreg*	*férgek*	worm	2	2	CVCVC	CVCCVC	3.26	.99	0.11	796.85	280.88
*horog*	*horgok*	hook	2	2	CVCVC	CVCCVC	2.69	.95	0.22	788.01	381.21
*piszok*	*piszkok*	dirt	2	2	CVCVC	CVCCVC	3.06	1.00	0.00	768.09	280.69
*pocok*	*pockok*	vole	2	2	CVCVC	CVCCVC	2.67	.99	0.11	784.51	341.33
*kéreg*	*kérgek*	bark	2	2	CVCVC	CVCCVC	3.07	.96	0.19	802.58	392.19
*kapor*	*kaprok*	dill	2	2	CVCVC	CVCCVC	2.75	.98	0.16	796.74	381.38
*szeder*	*szedrek*	blackberry	2	2	CVCVC	CVCCVC	2.66	.98	0.16	787.26	379.21
*bütyök*	*bütykök*	bunion	2	2	CVCVC	CVCCVC	2.71	.99	0.11	892.70	413.56
*alom*	*almok*	litter	2	2	VCVC	VCCVC	2.77	.91	0.29	822.03	328.88
*ajak*	*ajkak*	lip	2	2	VCVC	VCCVC	2.79	1.00	0.00	744.89	306.99
*iker*	*ikrek*	twin	2	2	VCVC	VCCVC	2.89	1.00	0.00	684.77	211.68
*torok*	*torkok*	throat	2	2	CVCVC	CVCCVC	2.89	.95	0.22	1062.09	543.16
*eper*	*eprek*	strawberry	2	2	VCVC	VCCVC	2.92	1.00	0.00	700.05	260.59
*burok*	*burkok*	shell	2	2	CVCVC	CVCCVC	2.93	.99	0.11	894.63	379.78
*retek*	*retkek*	radish	2	2	CVCVC	CVCCVC	2.94	.99	0.11	721.42	250.23
*agár*	*agarak*	greyhound	2	3	VCVC	VCVCVC	2.74	.80	0.40	894.16	334.06
*darázs*	*darazsak*	wasp	2	3	CVCVC	CVCVCVC	2.84	.98	0.16	717.75	250.42
*fazék*	*fazekak*	pot	2	3	CVCVC	CVCVCVC	2.87	.92	0.28	670.21	242.48
*fűz*	*füzek*	willow	1	2	CVC	CVCVC	2.96	.93	0.25	986.01	420.55
*fenék*	*fenekek*	bottom	2	3	CVCVC	CVCVCVC	3.00	1.00	0.00	806.34	272.20
*mocsár*	*mocsarak*	swamp	2	3	CVCVC	CVCVCVC	3.06	.98	0.16	789.51	216.15
*cserép*	*cserepek*	tile	2	3	CVCVC	CVCVCVC	3.18	.98	0.15	643.32	205.00
*gyökér*	*gyökerek*	root	2	3	CVCVC	CVCVCVC	3.22	1.00	0.00	766.51	226.24
*veréb*	*verebek*	sparrow	2	3	CVCVC	CVCVCVC	3.24	.98	0.16	650.58	199.94
*szekér*	*szekerek*	cart	2	3	CVCVC	CVCVCVC	3.34	1.00	0.00	704.29	320.65
*kanál*	*kanalak*	spoon	2	3	CVCVC	CVCVCVC	3.39	1.00	0.00	693.05	293.33

#### Past Tense Production Task

Present-tense 3^rd^ person singular verb forms are represented by the bare stem (in their indefinite indicative form). Past-tenses are formed by appending a –*t* or –*tt* suffix to the stem. For regulars, the suffix is always appended to the bare stem (with no phonological stem changes). Depending entirely on their phonology, the stems of regulars take either a –*t* affix or a –V*tt*, with the latter following vowel harmony (e.g., *csiszol—csiszolt*, polish—polished; *hámoz—hámozott*, peel—peeled); see Table 3. For irregulars, the stem undergoes idiosyncratic phonological stem changes (depending on stem class) as well as –V*tt* suffixation. The past tense production task includes two types of irregular stem changes. First, some Hungarian irregular verbs (so called sz-d-v- stems) end in –*szik* in its present 3^rd^ person indefinite indicative form. To form the past tense of these verbs, the –*szik* is deleted, and, after a stem change that is not entirely predictable, the –V*tt* is appended (e.g., *vastagszik—vastagodott*, thicken—thickened). For another class of irregular verbs, with so-called epenthetic stems, the past tense contains a stem form without the vowel in the final syllable, together with –V*tt* suffixation (e.g., *sajog—sajgott*, ache—ached) [32,33].

**Table 3:** Examples of Hungarian regular and irregular verb types used in this study, with 3^rd^ person singular present and past-tense forms.

	Stem type	Examples
Regular	Stem + -*t*	*csiszol—csiszolt*
		polish—polished
	Stem + V + -*tt*	*hámoz—hámozott*
		peel—peeled
Irregular	sz-d-v stem	*vastagszik—vastagodott*
		thicken—thickened
	epenthetic	*sajog—sajgott*
		ache—ached

The past tense production task consisted of 15 regular and 15 irregular verbs (see Table 4). All regular and irregular verbs ended in a consonant. The regulars and irregulars were matched pairwise on stem word length (2.67 vs. 2.67 syllables, respectively; *t* (14) < 0.001, *p* > .999) and CV (consonant vowel) structure (see Table 4), as well as natural logarithm-transformed bare stem frequency (2.77 vs. 2.76, respectively; *t* (14) = 0.43, *p* = .67) [34,35]. Inflected form frequencies were not available for all verbs, so regular and irregular forms were not matched on this factor. Each verb was pseudo-randomly ordered in the presentation list, with the different types evenly distributed throughout the list.

**Table 4:** Regular and irregular verbs in the past tense production task.

Stem form	Past tense form	Translation	Stem syllable #	Past tense syllable #	Stem CV structure	Past tense CV structure	Stem frequency (ln-transformed)	Mean Accuracy	SD Accuracy	Mean RT	SD RT
						**REGULAR**					
*vigasztal*	*vigasztalt*	comfort	3	3	CVCVCCVC	CVCVCCVCC	2.77	1.00	0.00	714.43	253.78
*továbbít*	*továbbított*	forward	3	4	CVCVCCVC	CVCVCCVCVCC	2.76	.99	0.11	621.46	178.54
*integet*	*integetett*	wave	3	4	VCCVCVC	VCCVCVCVCC	2.79	1.00	0.00	758.41	233.89
*érzékel*	*érzékelt*	perceive	3	3	VCCVCVC	VCCVCVCC	2.95	1.00	0.00	743.46	264.85
*nélkülöz*	*nélkülözött*	lack	3	4	CVCCVCVC	CVCCVCVCVCC	2.84	.99	0.11	745.99	315.56
*bírál*	*bírált*	criticize	2	2	CVCVC	CVCVCC	2.68	1.00	0.00	770.20	226.72
*csókol*	*csókolt*	kiss	2	2	CVCVC	CVCVCC	2.69	.99	0.11	704.09	224.81
*hódol*	*hódolt*	bow	2	2	CVCVC	CVCVCC	2.73	1.00	0.00	746.29	312.86
*kacag*	*kacagott*	laugh	2	3	CVCVC	CVCVCVCC	2.74	.99	0.11	683.00	233.05
*zokog*	*zokogott*	sob	2	3	CVCVC	CVCVCVCC	2.73	1.00	0.00	727.24	288.08
*lovagol*	*lovagolt*	ride	3	3	CVCVCVC	CVCVCVCC	2.68	.98	0.15	634.74	251.81
*birtokol*	*birtokolt*	own	3	3	CVCCVCVC	CVCCVCVCC	2.85	.96	0.19	744.52	294.27
*kárpótol*	*kárpótolt*	compensate	3	3	CVCCVCVC	CVCCVCVCC	2.84	1.00	0.00	650.61	264.33
*tanácsol*	*tanácsolt*	advise	3	3	CVCVCVC	CVCVCVCC	2.56	1.00	0.00	622.66	218.73
*mutogat*	*mutogatott*	point	3	4	CVCVCVC	CVCVCVCVCC	2.74	1.00	0.00	727.24	294.26
						**IRREGULAR**					
*gyanakszik*	*gyanakodott*	suspect	3	4	CVCVCCVC	CVCVCVCVCC	2.73	.94	0.25	826.96	398.81
*telepszik*	*telepedett*	settle	3	4	CVCVCCVC	CVCVCVCVCC	2.79	.98	0.16	775.04	332.25
*esküszik*	*esküdött*	swear	3	3	VCCVCVC	VCCVCVCC	2.88	.81	0.39	871.46	325.27
*öregszik*	*öregedett*	age	3	4	VCVCCVC	VCVCVCVCC	2.93	.98	0.16	776.41	276.48
*vetekszik*	*vetekedett*	rival	3	4	CVCVCCVC	CVCVCVCVCC	2.93	.99	0.11	943.59	409.07
*sajog*	*sajgott*	ache	2	2	CVCVC	CVCCVCC	2.56	.95	0.22	727.04	381.01
*rezeg*	*rezgett*	oscillate	2	2	CVCVC	CVCCVCC	2.73	.96	0.19	741.35	287.11
*kínoz*	*kínzott*	torture	2	2	CVCVC	CVCCVCC	2.75	.91	0.29	835.15	336.33
*jegyez*	*jegyzett*	mark	2	2	CVCVC	CVCCVCC	2.76	.98	0.16	897.68	459.59
*zörög*	*zörgött*	clatter	2	2	CVCVC	CVCCVCC	2.94	1.00	0.00	760.45	264.21
*párolog*	*párolgott*	evaporate	3	3	CVCVCVC	CVCVCCVCC	2.59	.99	0.11	773.70	242.38
*hömpölyög*	*hömpölygött*	roll	3	3	CVCCVCVC	CVCCVCCVCC	2.65	1.00	0.00	810.54	342.96
*tiszteleg*	*tisztelgett*	salute	3	3	CVCCVCVC	CVCCVCCVCC	2.66	1.00	0.00	719.82	264.53
*kavarog*	*kavargott*	swirl	3	3	CVCVCVC	CVCVCCVCC	2.74	1.00	0.00	659.96	252.44
*dübörög*	*dübörgött*	lumber	3	3	CVCVCVC	CVCVCCVCC	2.91	.98	0.16	785.13	349.97

### Procedure

Participants were tested individually using the same protocol as has been previously used in English [8,36,37]. The task was presented with E-Prime on a PC with Windows XP. For each item, the noun or verb stem was displayed (visually) alone, with a sentence just below it containing a blank to elicit the plural or past-tense form (e.g., *Ott vannak a* ___, There are the ___; *Tegnap ő* ___, Yesterday he ___). The item remained on the screen for a maximum of 10 seconds, or until the experimenter pressed the mouse button after the subject responded. In either case, the item was followed by a 750ms ISI indicated by a fixation cross. All prompt sentences were identical for plural elicitation, and likewise for past tense elicitation. Response time (RT) data were recorded via a microphone connected to a computerized timer, and were measured from the time the material appeared on the screen to the time the subject initiated their first response. Subjects were instructed to produce the missing form as quickly and accurately as possible based on the stem they had just seen. They were provided with 5 practice items prior to the beginning of each task.

### Analysis

Both accuracy and RTs were analyzed. During testing, the experimenter noted items where the RTs were not triggered by the subject’s response; these response times were not included in analyses. Accuracy analyses were performed on first responses; RT analyses were performed on correct first-responses. Very fast (< 200 ms) and slow RTs (> 3000 ms) were excluded from data analysis; these RTs constituted 1% of all correct first responses. Mixed-effect regression model analyses were conducted on both accuracy and log-transformed RT, with Regularity (regular vs. irregular) and Word Class (plural vs. past tense) as fixed factors, and Participants and Items as random factors [38,39]. The model was fitted using restricted maximum likelihood estimation (REML) for the continuous variable (RT). F-Test denominator degrees of freedom for both accuracy (logistic regression) and RT (linear regression) were estimated using the Kenward–Roger’s degrees of freedom adjustment to reduce the chances of Type I error [40]). Follow-up analyses were conducted with LSD post-hoc tests. All *p*-values are reported as two-tailed.

## Results

### Accuracy Analysis

**Fig 1:**
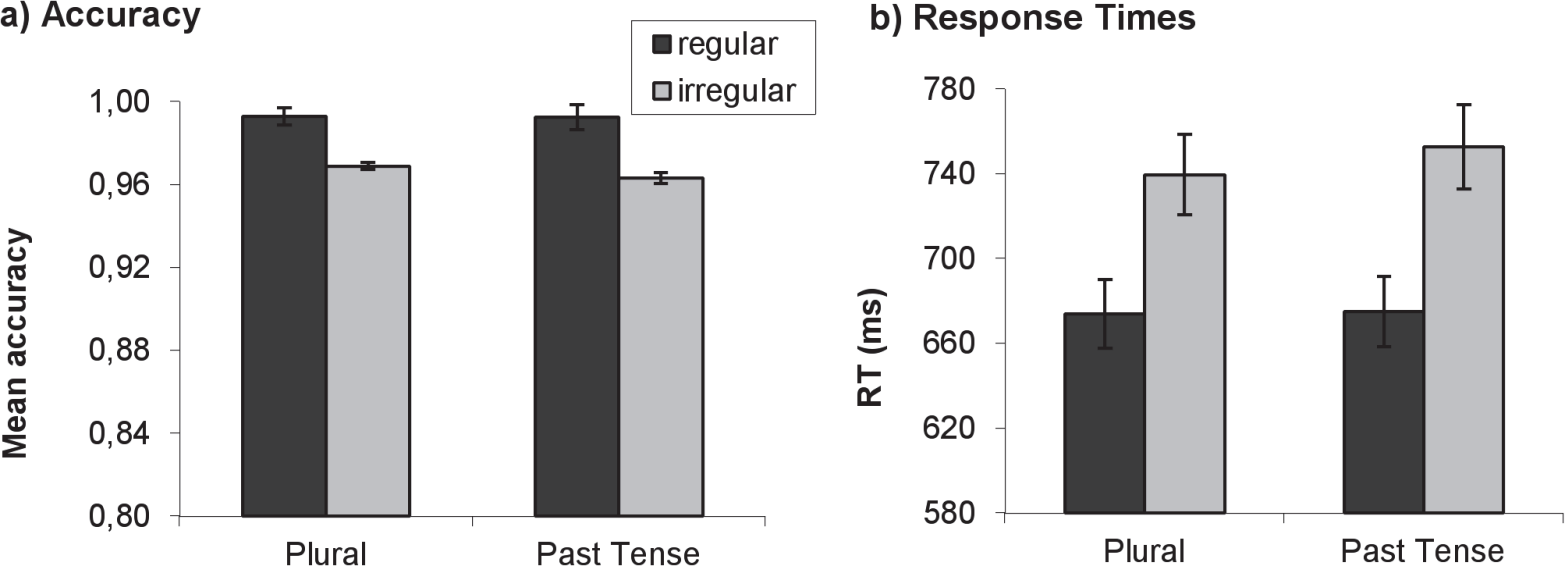
Mean accuracy (a) and mean response times (b) for the regular and irregular inflected forms of the plural and past tense production tasks. In both tasks participants were more accurate and faster at producing regular than irregular inflected forms. Error bars represent Standard Error of the Mean (SEM).

In the mixed effects regression model for accuracy, the maximal random effects structure justified by the data included random intercepts for Items, and by-participant random slopes for Regularity. Regularity (regular vs. irregular) significantly predicted accuracy (*F* (1, 84.99) = 10.99, *p* = .001), with participants less accurate at producing irregular than regular inflected forms (Fig. 1A). There was no difference between plural and past tense production (fixed effect of Word Class: *F* (1, 77.03) = 0.19, *p* = .66). Follow-up analyses confirmed that the regular advantage held for both plural and past tense production (*p*s < .02). The Word Class by Regularity interaction was not significant (*F* (1, 77.03) = 0.15, *p* = .70), suggesting that the regular advantage was to a similar extent in plural and past tense production.

### Response Time (RT) Analysis

In the mixed effects regression model for log-transformed RTs, the maximal random effects structure justified by the data included random intercepts for Participants and Items, and by-participant random slopes for Word Class., Regularity (regular vs. irregular) again significantly predicted RTs (*F* (1, 77.72) = 21.18, *p* < .001), with slower RTs in the production of irregular than regular inflected forms (Fig. 1B). There was no difference between plural and past tense production (fixed effect of Word Class: *F* (1, 128.96) = 0.03, *p* = .87). Follow-up analyses confirmed the regular advantage both for plural and past tense production (*p*s < .002). The Word Class by Regularity interaction was not significant (*F* (1, 77.72) = 0.26, *p* = .61).

## Discussion

The primary aim of the present study was to investigate the production of nominal and verbal inflectional morphology in a non-Indo-European agglutinative language with complex morphology, namely Hungarian. We tested healthy native speaking Hungarian adults on elicited plural and past tense production tasks, and recorded both accuracy and RT measures. Irregular inflected forms were produced both less accurately and more slowly than regulars in both plural and past tense production.

The greater accuracy observed for regulars than irregulars, for both nouns and verbs, is consistent with the accuracy advantage for regulars reported in most previous elicited production studies of inflectional morphology by healthy adults, including in the production of English past tenses [31,36,41,42,43,44,45,46] and plurals [46], Spanish present tenses [14] and past tenses [47], German past participles [48], and Hebrew plurals [49]. Note that no regular/irregular differences were reported in some studies, likely due to ceiling effects [17,50,51]. We are not aware of any production studies of healthy adults that have found worse performance on regular than irregular inflected forms.

The regular advantage observed here is also in line with the pattern found for the normal controls (and patients) in both previous studies of Hungarian plural production in adults [10,11] (the other two production studies examined children [28,30]). Note that in one of the two studies of adults both regular and irregular plurals were produced at ceiling by the controls [10], though not by the patients, who showed the expected pattern of worse performance on irregulars.

The response time pattern observed here of regulars being produced faster than irregulars, for both nouns and verbs, also replicates the RT pattern in most previous elicited production studies of healthy adults, including of English past tenses [41,44,46] and plurals [46], Spanish past tenses [15,47] and present tenses [14], German past participles [48,51] (in one study slower irregulars were found for lower but not higher frequency forms [51]), and Hebrew plurals [49]. Additionally, as with accuracy, some studies reported no response time differences between regular and irregular inflected forms, in the production of English past tenses [37,43,45], and higher frequency German past participles [51]. As indicated in the Introduction, response times have not previously been examined in inflected form production studies in Hungarian.

The pattern of results from this and previous studies may be most clearly interpreted within the context of (neuro)cognitive models of regular/irregular inflection. There are two broad classes of such models: dual system models and single mechanism models. According to dual system views, irregular forms crucially depend on memorized representations, with the exact nature of these representations varying across models, languages, and morphological systems [1,2,3,52,53]. On one view, irregular inflected forms may be stored as (structured or unstructured) wholes (e.g., *dug*), including even forms that appear to involve both a stem change and affix (e.g., *tengo*, (I) have in Spanish) [1,2,3,14,52]. Alternatively, the modified stem of irregulars may be stored, while the affix is attached with the same compositional mechanisms that apply to regulars (refs?). For example, the production of *tengo* from *tener* might involve the retrieval of *teng-* together with *o-*affixation by a separate mechanism. It has also been suggested that even stem changes in irregulars may in many cases depend on (phonological) rules, though crucially such rules must be linked in memory with particular stems due to the idiosyncratic nature of their application (e.g., *sing-sang*, *fling-flung*, *bring-brought*) [53]. In contrast, in dual systems models regulars are posited to be generally computed by a neurocognitive system that is distinct from the memory system in which representations of irregulars are stored; in particular this system is posited to underlie the composition of stems and affixes (eg, *walk* + -*ed*, *lány* + -*ok*) [1,2,3,52]. Note that on some dual system views regulars can also be memorized (e.g., as whole forms), with the likelihood of memorization a function of various factors [3,8,14,37,45]; e.g., some evidence suggests that higher frequency regulars tend to be stored [37,54].

According to single mechanism models, regular and irregular inflected forms are both computed by the same underlying computational mechanisms, which underlie a distributed associative memory [55,56,57,58,59]. In some single mechanism models the production of inflected forms depends on both phonological and semantic components within the associative memory, with regulars depending particularly on phonology (due to the consistency of the phonological mappings between stem and inflected forms), while irregulars rely more on semantics, by way of compensation due to the inconsistency of their phonological mappings [59].

Interpretation of the pattern of findings reported here and in previous studies differs somewhat between these models. According to dual system models, the depressed accuracy and slower response times of irregulars may largely reflect the difficulty of lexical retrieval, especially for less well learned items (e.g., those that are of lower frequency). This could explain production difficulties for irregular inflected forms no matter how they are represented in memory (e.g., as whole words, transformed stems, or links to phonological rules). In contrast, the knowledge of the rules that underlie regular transformations should be very well learned (because the rules apply across multiple words), so they should apply reliably and rapidly. In the present study neither the nouns nor the verbs were of very high frequency, underscoring the possibility of lexical retrieval difficulties for irregulars—as well as an absence of storage for regulars, consistent with reliable rule-production. Also consistent with this perspective, a previous study (see above) found that irregular forms were produced more slowly than regulars for lower but not for higher frequency items [51]—a pattern that may be explained not only by facilitated lexical access for higher frequency irregulars, but perhaps also by the storage of regulars alongside the irregulars. Finally, on a dual system view other factors might also contribute to worse performance producing irregular than regular inflected forms. For example, in the production of Hungarian plurals and past tenses (as well as in other languages and inflections, such as Spanish and Italian present tenses), irregulars may be less reliable and slower to produce than regulars if the former involve two or more steps (e.g., the retrieval of a transformed stem or the application of phonological rules, in addition to affixation), while regulars might involve only one (affixation)—at least for non-stem-changing regulars, which constituted all verbs and almost all nouns in the present study. Note that vowel harmony is a full predictable phonological process that affects regulars and irregulars alike. Thus overall, dual system models expect that regular inflected forms should be produced more reliably and faster than irregulars, as was observed both in the present study and in previous studies in a wide variety of languages.

Single mechanism models provide a different account [55,56,57,58,59]. Regular inflected forms should be produced relatively easily, and thus accurately and quickly, because of their consistent phonological mappings. In contrast, the less consistent phonological mappings of irregulars may lead to errors and slower processing. Thus, single mechanism models may also explain the pattern observed here in Hungarian, as well as previously in other languages.

In sum, this study replicates and extends findings from previous research examining the production of regular and irregular inflected forms in other languages. Most previous studies have investigated the regular/irregular distinction in Indo-European languages with relatively simple morphology. These have generally reported lower accuracy and slower response times for irregulars than for regulars. Here we show that a similar pattern is obtained in Hungarian, a non-European agglutinative language with complex morphology. Thus, overall, the data suggest that the production of irregular inflected forms is consistently more difficult than that of regulars, as measured by both accuracy and response times, across languages, language families, linguistic typologies, word classes, and types of irregular form (e.g., unaffixed such as *dug* or affixed such as *lovak*). Therefore the regular/irregular distinction appears to systematically correlate with a specific pattern of accuracy and response times across language types. This in turn suggests that further cross-linguistic as well as cross-methodological studies of this phenomenon are warranted.

Finally, we would like to emphasize that the present study is designed not only to examine the generalizability of previously observed regular/irregular patterns, but also to provide a useful carefully designed task to facilitate future research in Hungarian. Thus, we are making all items and item characteristics available to the reader (see Tables 2 and 4). We expect that these tasks and stimuli may be useful for a variety of future studies, for a variety of purposes. For example, their use with other methods, such as with fMRI, may help identify the neural correlates of regular and irregular nominal and verbal inflection in Hungarian, and thus help tease apart the competing theoretical accounts discussed above (e.g., by revealing which brain structures are differentially associated with the two morphological types). Additionally, the tasks and items may have translational impacts by providing useful tools for revealing the neurocognitive bases of language deficits in Hungarian patients with a variety of disorders in which regular/irregular distinctions have proved revealing in other languages, including Alzheimer’s, Parkinson’s, and Huntington’s diseases, aphasias, and neurodevelopmental disorders such as Specific Language Impairment, autism and Tourette syndrome [3,6,10,36,60,61,62].
